# The influence of preparation methodology on the concentrations of heavy metals in *Pleurozium schreberi* moss samples prior to use in active biomonitoring studies

**DOI:** 10.1007/s11356-020-11484-7

**Published:** 2020-11-08

**Authors:** Paweł Świsłowski, Grzegorz Kosior, Małgorzata Rajfur

**Affiliations:** Institute of Environmental Engineering and Biotechnology, University of Opole, B. Kominka 6a, 45-032 Opole, Poland

**Keywords:** Mosses, Biomonitoring, Heavy metals, Coefficient of variation, Research methodology, Air pollution

## Abstract

Active biomonitoring is used to assess environmental pollution of elements such as heavy metals by indicator species such as mosses. They are used, among others, in urbanized areas where no indicator species are found. In such study areas, mosses collected from sites considered to be ecologically clean shall be exposed. In this context, it is very important to prepare the mosses properly before the exposure, so that the information received about the condition of the environment is reliable. In 2018, studies were conducted in the forested areas of southern Poland—in Opolskie Province. *Pleurozium schreberi* mosses were used in these studies. Atomic absorption spectrometry with flame atomiser (F-AAS) was used to determine the concentrations of Mn, Fe, Ni, Cu, Zn and Pb present. The aim was to study the influence of preparation methodology on *Pleurozium schreberi* moss samples prior to use in active biomonitoring studies. Four different methodologies were tested across four different sample locations (with varying levels of pollution). The results of the research were analysed and the coefficient of variation (*CV*) was determined. The value of the *CV* is influenced, among other things, by the location of the particular sample and the level of pollution by, for example heavy metals, in the moss. The research conducted proves that of the four methods used to prepare mosses for later exposure in active biomonitoring, the best method is averaging with simultaneous conditioning of mosses in demineralised water. This treatment causes the *CV* coefficient to fall below 10% for most of the metals determined in the moss samples. It has also been shown that maintaining moss collection methodology in accordance with ICP Vegetation standards (open/wooded area—tree canopy) also has a significant impact on the result obtained. Statistical analysis confirmed (Wilcoxon test) that the method of processing the mosses significantly influenced the results obtained. Thanks to the appropriate preparation of the mosses before exposition, they can be used in active biomonitoring of, for example, urban areas.

## Introduction

Various plant and animal species have been used in studies on monitoring air, water or soil quality (de Oliveira et al. 2016; Oishi 2018). Mosses are commonly used biomonitors to assess atmospheric aerosols (Kosior et al. 2010; Korzeniowska and Panek 2012). They are regarded as one of the main bioindicators of air pollution (Salo and Mäkinen 2014). Mosses do not have a root system and absorb nutrients and pollutants through their surfaces as a result of wet or dry deposition (Ares et al. 2012). For example, ectohydric moss *Pleurozium schreberi* has been used extensively throughout the European Union (Lequy et al. 2017; Nickel and Schröder 2017), including in Poland (Olszowski et al. 2012; Rajfur et al. 2018).

Several aspects need to be considered when analysing biomonitoring studies using mosses. One may draw different conclusions, depending on the type of study (active or passive biomonitoring) and the method of preparation.

Passive biomonitoring is a useful method for air quality assessment, including that of concentrations of heavy metals in the atmosphere. The technique enables the qualitative comparison of polluted and pollution-free areas in order to identify precisely the sources and scale of atmospheric aerosol pollution (Fernández et al. 2015). Passive biomonitoring studies are not fully standardized; there is still the question of the mass of a given sample and the number of subsamples. In the case of passive biomonitoring, the problem of measurement uncertainty occurs quite frequently and is related to the applied protocol/method for preparing samples for analysis (Fernández et al. 2015; Aboal et al. 2017). Examples of pollution monitoring studies of heavy metals or radionuclides indicate the heterogeneity of samples and, therefore, the inability to compare them using, for example, two moss species (Krmar et al. 2013; Boquete et al. 2014). The authors recommend the use only of the green parts of mosses in this type of biomonitoring (Dmuchowski et al. 2011; Boquete et al. 2014). A comparison of various bioindication methods showed that *P. schreberi* moss delivered distinctly different results from other mosses, and this method did not offer the highest correlation coefficient compared with other methods (Dmuchowski et al. 2011). A comparison of two biomonitoring methods (active and passive) proves that the former offers more advantages due to the standardization of the material (Fernández et al. 2000).

The second type of biomonitoring studies using mosses is active biomonitoring, the so-called moss bag method. It is very simple to carry out, reliable and financially acceptable due to the low cost of material acquisition (Debén et al. 2018). The method is used for the simultaneous monitoring of concentrations of many environmentally importance analytes. Active monitoring is commonly used throughout Europe to assess pollution associated with heavy metals, polycyclic aromatic hydrocarbons (PAHs) or other organic pollutants (Aničić et al. 2009; Vuković et al. 2015a; Kosior et al. 2017). The method is used both in urban and industrial areas (Capozzi et al. 2016). However, the implementation of such studies requires the systematization of experiment procedure. All studies which use the moss bag method emphasize the element of the standardization of the research protocols and procedures for preparing moss samples prior to exposure in the field (Iodice et al. 2016; Salo et al. 2016; Arndt and Planer-Friedrich 2018).

Preparation of the material most often includes collecting mosses from relatively clean areas and removing all impurities (soil, leaves, needles, etc.) (Vuković et al. 2015a; Di Palma et al. 2017), as well as rinsing with water prior to exposition in bags/sachets (Giordano et al. 2013; Varela et al. 2016). This is done in order better to clean the material and remove any plant remains or soil particles (Ares et al. 2014), but mainly in order to maintain the properly low initial level of trace elements in the mosses prior to exposition. The study results confirm the importance of the washing procedure prior to the use of the material in this field of study, because the analysis of the washing solutions demonstrates that the concentration of the analytes decreases considerably (Calabrese et al. 2015). The study results indicate a higher content of heavy metals in the ‘wet’ moss bags after exposition compared with those that were not conditioned in water. The research methodology often includes mixing samples prior to direct exposition, in order to obtain homogeneous material (Ares et al. 2015; Vuković et al. 2015b).

Based on literature sources, a study into the influence of the method of preparing biological material on the distribution of heavy metal concentration in mosses was conducted.

The objective of the research was to assess the biological material homogeneity, based on the method of preparation, for subsequent exposition within active biomonitoring of, for example, urbanized city areas. The data obtained enabled the establishment of standardized procedures for preparing mosses for exposition.

## Materials and methods

### Materials

**Fig. 1:**
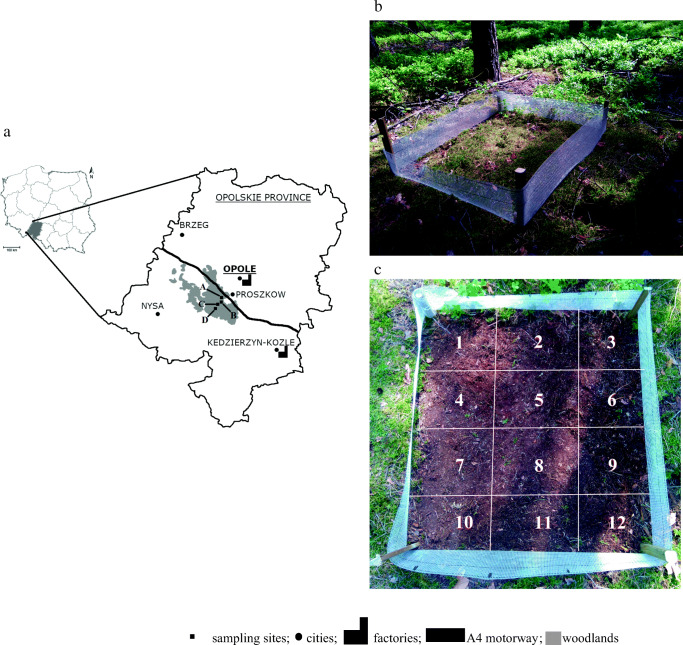
Locations of measuring points (**a**) with image one of the study surface (**b**) and 12 points marked in the surface area (**c**)

*Pleurozium schreberi* mosses were used in the research. This is a species commonly found in Europe, including in Poland, which is used as an air quality bioindicator and also in active biomonitoring (Viskari et al. 1997; Suoranta et al. 2016; Boquete et al. 2017; Mahapatra et al. 2019). Mosses taken from the natural environment have been used in the study because, thus far, no methodology has been developed and no *Pleurozium schreberi* clones have been grown (the authors of the publication make such attempts). Mosses were collected in the Niemodlinskie Forest in Proszkow Forest Region in Poland’s Opolskie Province. Samples of mosses were collected in four locations, marked A, B, C and D (Table 1; Fig. 1). The availability of unpolluted anthropogenic sites from which mosses can be collected without endangering the native population is limited. For this purpose, specimens taken from sampling points with different levels of heavy metal contamination were used for comparative testing.

**Table 1:** Measuring points

Point	GPS track	Description
A	50° 35′ 14″ N 17° 48′ 52″ E	Woodland adjacent to the A4 motorway (100 m from motorway)
B	50° 35′ 09″ N 17° 48′ 49″ E	Location near a forest road (300 m from motorway)
C	50° 34′ 53″ N 17° 48′ 31″ E	Location selected at random in deep woodland (900 m from motorway)
D	50° 34′ 53″ N 17° 48′ 22″ E	Open area selected as a reference point, in line with the recommendations of ICP Vegetation (ICP Vegetation 2020) (1 km from motorway)

The material collection was carried out along a total length of approximately 1 km—from an area adjacent to the A4 motorway to the last point, D (Table 1). The described areas is under the administration of Proszkow Forest Region—Niemodlinskie Forest (Fig. 1a) and has a majority of coniferous trees.

Moss samples were collected from an area of 1 m × 1 m at each point. Within the 1 m^2^ (Fig. 1b), 12 points were marked from which mosses were collected for study (Fig. 1c). Collection of the material was carried out in spring (April) 2018. The collected moss samples were taken to a laboratory and dried at room temperature until dry mass (d.m.) was obtained. Next, the green part of the gametophyte, live and active tissues (Boquete et al. 2014), was separated from the moss to be used in the research (Jiang et al. 2018; ICP Vegetation 2020). Next, the mosses were prepared according to the methods described in Table 2.

**Table 2:** Methodology of moss sample preparation

Method	Description
1	Removal of adhered materials/impurities (e.g. leaves, needles, soil)
2	As above + conditioning mosses in demineralised water (each of 12 samples with a mass of 0.6 g d.m. was conditioned and left in 1 l of demineralised water with conductivity of κ = 0.5 μS/cm for 1 h)
3	As in method 1 + averaging (manually mixing up the 12 moss samples taken from one measuring point and then separating them again)
4	As above + conditioning in 12 L of demineralised water with conductivity of κ = 0.5 μS/cm for 1 h

### Methods

Mosses from measurement points A, B and C were prepared by four different methods. Table 2 presents the assumptions of the methods of moss preparation prior to determining the concentrations of heavy metals. For each method, 12 moss samples (*n* = 12) were taken. A total of 144 samples were analysed from points A, B and C. Since measurement points C and D were only 100 m apart and were less contaminated with heavy metals, the mosses taken from point D were prepared only by method 1 (12 samples). In total, 156 moss samples were prepared and analysed.

Before mineralisation, the moss samples were dried at room temperature until dry mass was obtained. Each moss sample with a mass of 0.400 ± 0.001 g d.m. was prepared in this way and mineralised in a mixture of nitric acid (V) and hydrogen peroxide (HNO_3_ 65%:H_2_O_2_ 37% = 5:3) using a Speedwave Four Berghof DE microwave oven. The mineralisation process was carried out at a temperature of 180 °C.

The concentrations of heavy metals (Mn, Fe, Ni, Cu, Zn and Pb) were determined using an atomic absorption flame spectrometer (F-AAS) type iCE 3500 (series 3000) made by Thermo Scientific, USA.

### Quality control

Table 3 presents the instrumental detection limits (*IDL*) and instrumental quantification limits (*IQL*) of the iCE 3500 spectrometer. The results were converted for 1 kg of sample. The calibration of the spectrometer was performed with a standard solution from ANALYTIKA Ltd. (CZ). The values of the highest concentrations of the models used for calibration (7.5 mg/dm^3^ for Mn, 10 mg/dm^3^ for Fe, 5 mg/dm^3^ for Ni, Cu, Zn, Pb) were approved as linear limits to signal dependence on concentration. The concentrations of metals were determined in solution after mineralisation and dilution and filtration into 25-cm^3^ volumetric flasks.

**Table 3:** The instrumental detection limits (*IDL*) and instrumental quantification limits (*IQL*) for the iCE 3500 (mg/dm^3^) spectrometer (Thermo Fisher Scientific Inc. 2011)

Metal	*IDL*	*IQL*
Mn	0.0016	0.020
Fe	0.0043	0.050
Ni	0.0043	0.050
Cu	0.0045	0.033
Zn	0.0033	0.010
Pb	0.0130	0.070

Table 4 shows the concentrations of heavy metals in certified reference materials BCR-482 *lichen*, produced at the Institute for Reference Materials and Measurements, Belgium.

**Table 4:** Comparison of measured and certified concentrations in BCR-482 *lichen*

Metal	BCR-482 *lichen*		AAS (*n* = 5)		Dev.**
	Concentration	Measurement uncertainty	Average	± SD* of the concentrations	
	(mg/kg d.m.)				(%)
Mn	33.0	0.50	31.7	0.68	− 3.90
Fe	804	160	771	154	− 4.10
Ni	2.47	0.07	2.16	0.32	− 13.0
Cu	7.03	0.19	6.63	0.17	− 5.70
Zn	100.6	2.20	95.1	2.30	− 5.50
Pb	40.9	1.40	38.2	1.00	− 6.60

*Standard deviation

**Relative difference between the measured (*c*_z_) and certified (*c*_c_) concentration 100%∙(*c*_z_ − *c*_c_)/*c*_c_

Table 5 presents the method detection limit (*MDL*) and the method quantification limit (*MQL*) of the AAS analytical method, determined on the basis of the results of metal determination in mineralised moss samples, so that the concentration of metal values (*C*_M_) would be comparable with the *IQL* values. The *IQL* value for each of the quantified metals was accepted as the expected detection limit of the analytical method. Twelve determinations were carried out, one for each metal, in line with the analytical procedure. *MDL* and *MQL* values were determined based on own research presented in this article and collected in the following table.

**Table 5:** Parameters of the heavy metal determination method in moss samples (mg/dm^3^)

Metal	Mn	Fe	Ni	Cu	Zn	Pb
*MDL*	0.891	1.00	0.057	0.015	0.120	0.026
*MQL*	2.67	3.01	0.172	0.044	0.359	0.079

In order to assess the relative differentiation of the results of the concentration levels (mg/kg d.m.) of analytes in the mosses collected from the studied area, the *CV* was determined, which refers the value of standard deviation *s* (absolute differentiation of the feature distribution) to the mean value of *x*_*av*_ (Konieczka and Namieśnik 2018):

1 $$ CV=s/{x}_{av}\cdotp 100\ \left(\%\right) $$

The results were interpreted based on this coefficient, which is frequently used in analysis of biomonitoring studies ((Fernández et al. 2015; Dołęgowska 2016; Zhou et al. 2017).

The Lilliefors modifications of the Kolmogorov-Smirnov test failed to prove a hypothesis about data normality (Zar 2010). Therefore, differences between the preparation methods in terms of concentrations of elements in the mosses were evaluated by the Wilcoxon test. All calculations were carried out using Statistica 13 software (StatSoft Inc 2017).

## Results and discussion

**Fig. 2:**
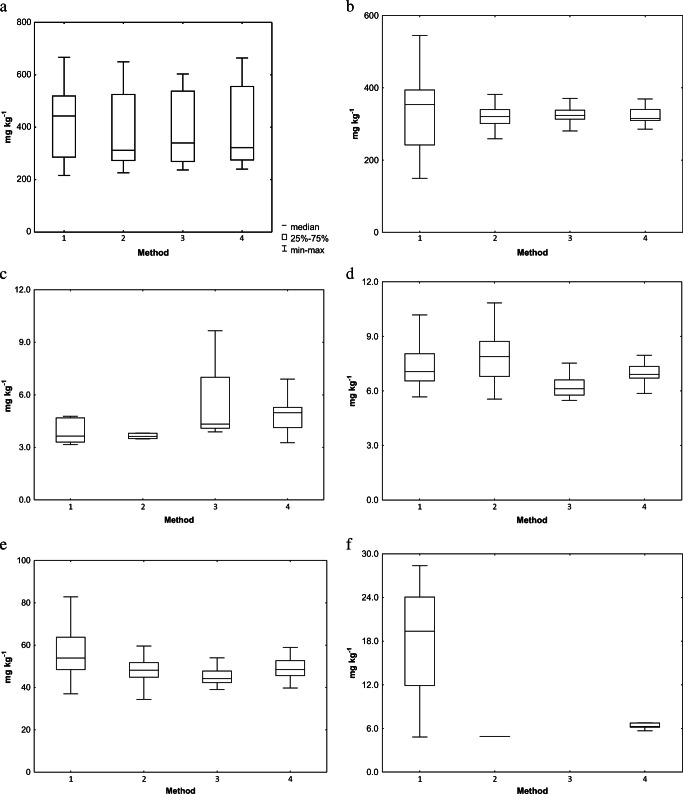
Comparison of heavy metal concentrations. **a** Mn, **b** Fe, **c** Ni, **d** Cu, **e** Zn, and **f** Pb in moss samples depending on method

Figure 2 a–f show an analysis of the concentrations of elements in mosses, depending on the method applied, and regardless of the area type from which the mosses were collected.

On the basis of the results of the Wilcoxon test, it can be stated that for manganese the preparation method does not significantly influence changes in concentration distribution of this analyte. In the case of iron, considerable differences can be noticed between concentration distribution for method 1, compared with methods 2, 3 and 4. This was confirmed by statistical calculations using the Wilcoxon test (Table 6). The data presented in Table 6 confirms that appropriate preparation of the material influences the quality of the result, where statistically significant differences between methods occur. In the case of copper, it can be observed that the lowest fluctuation of results was obtained when the moss was prepared using methods 3 and 4. Zinc is a similar case where, for methods 2, 3 and 4, considerable differences can be observed between the concentration distribution of the element, compared with method 1. Similarly, as above, this is reflected in the statistical data collected in Table 6. In the case of lead, there are considerable differences between the first (simplest) method of sample preparation and the relatively small distribution of data in method 4.

**Table 6:** The results of the Wilcoxon test for heavy metals depending on method

v1	v2	*W*	*p*
Mn1	Mn2	290.000	0.509
Mn1	Mn3	277.000	0.388
Mn1	Mn4	260.000	0.258
Fe1	Fe2	659.000	***
Fe1	Fe3	618.000	***
Fe1	Fe4	633.000	***
Ni1	Ni4	89.000	0.235
Cu1	Cu2	269.000	0.323
Cu1	Cu3	560.000	***
Cu1	Cu4	494.500	*
Zn1	Zn2	521.000	**
Zn1	Zn3	608.000	***
Zn1	Zn4	465.000	*
Pb1	Pb4	44.000	**

v1, v2—value 1 and value 2; *W*—test statistical value/the sum of the signed ranks; *p* value—probability value/significant level alpha; Mn1—method 1 for Mn; Mn2—method 2 for Mn; Mn3—method 3 for Mn; Mn4—method 4 for Mn

* < 0.05; ** < 0.01; *** < 0.001

The mosses collected from area C had the highest average for manganese and iron. This effect may be caused by the addition of more of these elements to mosses due to the rainfall under tree crowns and leaching from tree trunks (Krawczyk et al. 2009). Cadmium was not determined in any preparation method—it was below the quantification limits of the applied analytical method. The distribution of lead may be influenced by the polluting source as, for example, in area A—traffic (Singh et al. 2017), and its concentration in method 4, and the statistically relevant difference is the result of the appropriate preparation and homogenisation of the material, which enabled its determination (Fernández et al. 2015).

The coefficient of variation values was determined in the next stage of the results analysis. A comparison was carried out of the difference in heavy metals concentration values determined in the samples of moss, prepared for analysis under the four proposed methods (Table 7).

**Table 7:** Results of the coefficient of variation from areas A, B and C (%)

Metal	1	2	3	4
A area—method				
Mn	16.1	13.7	*5.43*	5.84
Fe	17.3	9.72	*3.99*	5.99
Ni	4.71	4.65	-*	24.1
Cu	5.35	18.0	7.41	*4.36*
Zn	7.59	7.77	6.52	*4.57*
Pb	-*	-*	-*	*8.76*
B area				
Mn	10.6	8.31	*5.38*	5.84
Fe	8.44	7.57	3.68	*2.86*
Ni	54.6	-*	-*	*14.6*
Cu	40.0	15.3	50.3	*6.34*
Zn	10.9	14.4	7.96	*7.38*
Pb	-*	-*	-*	*20.0*
C area				
Mn	9.37	9.02	*4.91*	7.70
Fe	11.5	9.75	*4.24*	5.63
Ni	44.4	-*	*6.28*	29.8
Cu	35.1	22.2	9.11	*7.77*
Zn	25.8	16.4	8.86	*7.11*
Pb	-*	-*	-*	*51.6*

Italicized values mean the lowest value

*Below the limit of quantification (mg/kg d.m)

On the basis of the research undertaken, it should be stated that the material averaging and conditioning method (method 4) proved to be the best method for preparing moss samples prior to exposition in future active biomonitoring. This is because mixing different samples of moss (mixing subsamples collected in sampling site) produces more averaged material. It has been stated that the heterogeneity of the material is caused mainly by the sediment of various sized dust particles on the surface of the mosses, which influences the variability of samples as far as the heavy metal pollution level is concerned (Aboal et al. 2008). By conditioning samples in demineralised water, we remove dust particles from the surface and eliminate the influence of soil particles on the content of certain elements in mosses (Fernández et al. 2010), thanks to which we receive a purer sample (without ions of elements in ion-exchange centres) ready for exposition in, for example, urbanized areas. This is of key importance because improperly prepared moss samples lead to false results, wrong interpretations and therefore inappropriate conclusions.

Further methodological studies looked at the influence of the area from which the sample is collected on the level of heavy metals pollution. The conclusion was based on the assumptions and recommendations of the ICP Vegetation programme, which presents, among other things, the method and location from which moss samples should be collected for the purpose of research. While focusing on these recommendations, an attempt was made to analyse within our own study, how material averaging changes between mosses collected from an open area without trees in comparison with samples collected from area C—deep in the woods, under trees.

Comparing the value of the coefficient of variation for the biological material prepared for analysis in line with method 1 for the two areas, it should be stated as follows: the *CV* was lower for area D than for area C (Table 8). However, it should be emphasized that, for the samples collected from area D, it was also possible to determine the *CV* for lead (based on the determined concentration). In addition, it was only possible to determine the concentration of Ni and calculate its coefficient of variation from the samples collected from area C. These results confirm the assumptions of the ICP Vegetation programme, as regards the method of sample collection, recommending open spaces, preferably without trees. Therefore, it is important to select carefully the locations for sampling material for research. As illustrated in Table 8 above, it later turns out to be of key importance for the quality of the result obtained. This is also important because many publications do not include a detailed description of the sample-taking location, or samples are collected from inappropriate locations, without using any of the protocols or recommendations that have been set in moss sample-taking methodology.

**Table 8:** A comparison of *CV* for mosses collected from areas C and D and prepared for analysis in accordance with method 1 (%)

Metal	C area	D area
Mn	9.37	*6.02*
Fe	11.5	*7.59*
Ni	44.4	-*
Cu	35.1	*6.24*
Zn	25.8	*15.4*
Pb	-*	54.3

Italicized values mean the lowest value

*Below the limit of quantification (mg/kg d.m)

## Conclusions

Biomonitoring offers a cheap and simple supplement to the classical methods for assessing environmental quality and condition. However, for biomonitoring to be comparable and of equal quality to these methods, it is very important to prepare material properly prior to exposition, particularly when conducting active biomonitoring. This information is often missing from the scientific literature, or the descriptions provided in the sections on materials and methods are superficial.

This study achieved its goal by showing that the obtained result and the range of variation of biological material are influenced not only by the proper selection of the location for moss-sampling but also by the processing method used prior to exposition in, for example, urbanized areas. The study describes four methods of preparing moss for exposition. The results of the laboratory research undertaken indicate that, of the four applied methods, the best one is averaging with simultaneous conditioning of mosses in demineralised water. This procedure results in the decrease of *CV* to below 10% for most metals determined in moss samples. The analysis of the results with the Wilcoxon test confirmed statistically significant differences between the methods used for most metals and showed that appropriate moss preparations increase the homogeneity of samples.

It was also determined that applying moss sample collection methodology in line with ICP Vegetation (open area/trees, crowns) has a significant influence on the result obtained.

## Data Availability

All data generated and analysed during this study are available from the corresponding author on reasonable request.
